# Association between Thrombophilia Gene Polymorphisms and Preeclampsia: A Meta-Analysis

**DOI:** 10.1371/journal.pone.0100789

**Published:** 2014-06-26

**Authors:** Xi Wang, Tingting Bai, Shengnan Liu, Hong Pan, Binbin Wang

**Affiliations:** 1 Graduate School of Peking Union Medical College, Beijing, China; 2 National Research Institute for Family Planning, Beijing, China; IPO, Inst Port Oncology, Portugal

## Abstract

**Objective:**

To estimate the relationship between the risk of preeclampsia and two thrombophilia gene single-nucleotide polymorphisms (SNPs), the factor V G1691A SNP and the prothrombin G20210A SNP.

**Date Sources:**

A systematic search of the English-language literature up to November 2012 was performed using Medline and EMBASE. Search terms included “preeclampsia,” “thrombophilia,” “factor V Leiden,” “prothrombin gene 20210,” and their combinations.

**Result(s):**

Thirty-seven studies with 5048 preeclampsia patients and 6796 controls were included in the meta-analysis. We found that the prothrombin G20210A polymorphism was associated with an increased risk of all preeclampsia (pooled odds ratio (OR) = 1.81, 95% confidence interval (CI) 1.25–2.63) and severe preeclampsia (pooled OR = 3.02, 95%CI 2.06–4.45). Meanwhile, the pooled OR for the association between factor V Leiden and all preeclampsia was 1.60 (95%CI 1.28–2.00) and 2.45 (95%CI 1.63–3.69) for the cases of severe preeclampsia.

**Conclusion(s):**

This meta-analysis supports that the factor V G1691A SNP and the prothrombin G20210A SNP are associated with an increased risk for both preeclampsia overall and severe preeclampsia.

## Introduction

Preeclampsia (PE), a complex disorder of pregnancy, is a major cause of maternal mortality worldwide, and is responsible for 15% to 20% of maternal mortality in developed countries [1]. It is thought to be a heterogeneous syndrome defined clinically by increased maternal blood pressure and proteinuria occurring after 20 weeks of gestation. The clinical manifestation of preeclampsia varies widely, from mild preeclampsia with only a moderate increase in blood pressure and proteinuria, to the most severe disorder with seizures and HELLP (hemolysis, elevated liver enzymes, and low platelets) syndrome, which greatly threatens the lives of pregnant women and their fetuses [2].

Despite many studies conducted previously, the etiology of PE remains unclear. Many hypotheses have been presented to explain the development of PE, including maternal and fatal genetic and environmental factors, classified into four categories: 1) immune maladaptation, 2) placental ischemia, 3) oxidative stress, and 4) genetic susceptibility [3]. Combinations of the many interactions between these categories may contribute to the etiology of preeclampsia. Among these, hypercoagulability, which plays a potential role in preeclampsia development, leads to decreased placental blood perfusion caused by microthrombi in placental blood vessels, eventually resulting in placental ischemia [4]. In the 1990s, factor V G1691A (also known as factor V Leiden, FVL) [5] and prothrombin G20210A (PT 20210) [6] were first reported to be related to a hypercoagulable state. Factor V 1691A allele and prothrombin 20210A allele were identified as risk factors for venous and arterial thrombosis. From then on these two genetic polymorphisms were inferred to be associated with PE and many studies have been conducted to explore the relationship between them. Several studies suggested that FVL [7]–[9] and PT 20210 polymorphism [9], [10] were associated with an increased risk of preeclampsia. However, other studies showed inconsistent results.

These inconsistent findings may be due to the differences in sample size, study design, ethnicity, and other aspects [11]. A meta-analysis is a useful tool with which to overcome these obstacles, owing to its increased statistical power. A previous attempt [12] has been made to explore the association between these two polymorphisms and the susceptibility of preeclampsia using a meta-analysis that included a relatively small number of studies. We therefore performed a meta-analysis to make a more precise assessment by adding more studies implemented in recent years. Furthermore, an evaluation of the relationship between these two SNPs and severe PE was conducted.

## Materials and Methods

### Search strategy

A computerized search of the English-language literature up to November 2012 was performed using Medline and EMBASE. We used the following search terms and the combinations thereof: “preeclampsia,” “thrombophilia,” “factor V Leiden,” and “prothrombin gene 20210.” The inclusion and exclusion criteria were as follows: (a) published studies with full text about the relationship between FVL or prothrombin G20210A and preeclampsia; (b) case-control studies; (c) studies with sufficient data to calculate odds ratio (OR) and 95% confidence interval (CI); (d) Reviews, letters, and single case reports were excluded; (e) if one group of patients was used in two or more studies, the study with the largest number of patients was selected.

### Data extraction

Two investigators performed the literature search and screened the studies independently according to the inclusion and exclusion criteria. Agreements were reached after discussions if discrepancies occurred.

When all the eligible studies were included, we extracted the following information for each study: first author’s name, year of publication, study design, country, ethnicity (Caucasian, Asian or other ethnicities), and number of cases and controls for each FVL or prothrombin gene polymorphism genotype. In terms of disease definition, the criteria of the American College of Obstetricians and Gynecologists [13] and the National High Blood Pressure Education Program Working Group Report on High Blood Pressure in Pregnancy [14] were mostly used, in 13 [10], [15]–[26] and 6 [9], [27]–[31] studies, respectively, among which the definitions of preeclampsia and severe preeclampsia varied little. In these studies, preeclampsia was defined as systolic blood pressure ≥140 mmHg and diastolic blood pressure ≥90 mmHg, with the presence of proteinuria by 24-h urinary excretion exceeding 300 mg after 20 weeks of gestation. Severe preeclampsia was determined if one or more of the following features were present: blood pressure ≥160/110 mmHg; proteinuria >3+; oliguria; seizures; visual disturbances; headache; HELLP (hemolysis, elevated liver enzymes, and low platelets); right upper-quadrant pain; and thrombocytopenia. The remaining studies [7], [8], [32]–[52] used modified but relatively compatible diagnostic criteria.

In several studies, the authors classified the patients into two categories according to the severity of disease: severe PE group and not severe PE group while only severe preeclampsia patients were recruited into case group in some other studies. However, no similar classification was made in the remaining studies, in which each individual diagnosed as preeclampsia (not necessarily as severe preeclampsia) was included. To explore whether genetic thrombophilias increase the risk for preeclampsia in general or only for severe preeclampsia, we conducted two separate analyses: one involving all patients with preeclampsia (severe and not severe), and another separate analysis of severe preeclampsia in those studies that identified these cases separately.

### Statistical analysis

Heterogeneity among studies was measured by the *Q*-statistic test and the *I*-squared statistical test. A fixed-effect model using the Mantel-Haenszel method was applied to calculate the pooled OR and 95% confident intervals (95%CI) if no or small heterogeneity was found. Otherwise a random-effect model using the DerSimonian-Laird method was used. Meta-regression was conducted to explore the source of heterogeneity, and influence analysis was performed when necessary. Studies with any zero cells were adjusted by adding 0.5 to each cell of the 2×2 table. Publication bias was assessed by funnel plot and Egger’s test. All analyses were performed using Stata 11.0 software (StataCorp LP, College Station, TX, USA).

## Results

**Figure 1:**
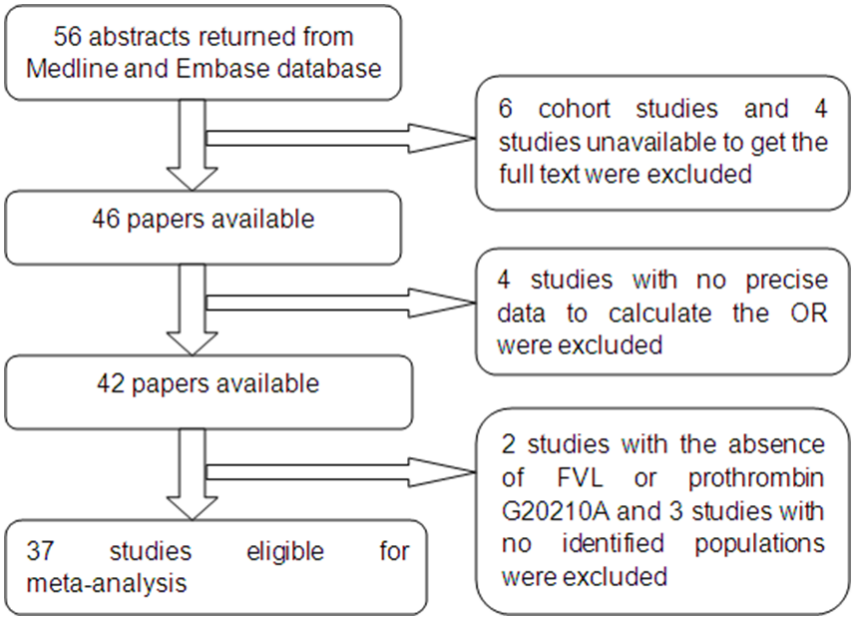
Flow diagram of meta-analysis literature search results.

Based on our search criteria, a total of 42 studies were included in this analysis. Two studies [28], [36] were excluded because of the absence of FVL in either patient or control groups, one of which identified this SNP in a Japanese population[28]. With regard to ethnic background, it was difficult to identify the ethnic origin of individuals in three studies [24], [31], [34], which were therefore excluded. Ultimately 37 studies were included in our meta-analysis, with 23 studies evaluating the PT 20210 polymorphism and 35 analyzing the FVL. The study selection process is presented in Figure 1. In terms of study design, eight studies [21], [26], [37]–[42] were prospectively designed and the rest were retrospective case-control studies. The data of each group are shown in the tables. In these studies, homozygotes of factor V 1691A allele and prothrombin 20210A allele were rarely detected in either the patient or the control groups, so the heterozygotes and homozygotes of each thrombophilia gene mutation were considered together. Table 1 and 2 present the results of studies that include cases of preeclampsia (not necessarily defined as severe), and Table 3 and 4 show the studies that include cases identified as severe preeclampsia. In twelve studies [15]–[17], [22], [25], [37]–[40], [48], [49], [52], only severe preeclampsia was defined and in seven[9], [10], [18], [32]–[35], the authors defined both preeclampsia and severe preeclampsia and performed further analysis of the association between the two polymorphisms and severe preeclampsia after analysis for all preeclampsia. In the remaining studies, the authors only defined preeclampsia and didn’t describe the distribution of mild and severe cases. However, in Mello’s study [10], which included 402 mild PE cases and 406 severe PE cases, the investigators analyzed mild PE and severe PE separately because of the high percentage of patients with severe preeclampsia, which did not reflect the prevalence of the disease in the general population.

**Table 1:** Case-control studies of Prothrombin 20120 G-A Polymorphism and All Preeclampsia.

study	country	ethnicity	cases		controls		OR	95%CI
			GG	GA/AA	GG	GA/AA		
De Groot(1999)	Netherland	Caucasian	158	5	157	6	0.83	(0.25–2.77)
Kupferminc(2000)	Israel	Caucasian	73	7	151	5	2.90	(0.90–9.44)
Higgins(2000)	Australia	Caucasian	134	5	116	3	1.44	(0.34–6.17)
D’Elia(2002)	Italy	Caucasian	57	1	73	1	1.28	(0.08–20.92)
Morrison(2002)	Scotland	Caucasian	396	4	164	0	3.73	(0.20–69.74)
Prochazk(2003)	Czech	Caucasian	37	1	50	0	4.04	(0.16–102.0)
Fabbro(2003)	Italy	Caucasian	50	2	78	2	1.56	(0.21–11.43)
Driul(2005)	Italy	Caucasian	38	1	63	1	1.66	(0.10–27.29)
Mello(2005)	Italy	Caucasian	386	16	397	5	3.29	(1.19–9.07)
Demir(2006)	Turkey	Caucasian	65	5	95	7	1.04	(0.32–3.43)
Yalinkaya(2006)	Turkey	Caucasian	96	4	98	2	2.04	(0.36–11.41)
Hiltunen(2009)	Finland	Caucasian	244	4	673	6	1.84	(0.52–6.57)
Best(2009)	US	American Indian	86	1	164	1	1.91	(0.12–30.86)
Kahn(2009)	Canada	Caucasian	110	3	435	8	1.48	(0.39–5.68)
Seremak-Mrozikiewicz(2010)	Poland	Caucasian	104	5	395	5	3.80	(1.08–13.37)
Malek-Khosravi(2012)	Western Iran	Caucasian	194	4	98	3	0.67	(0.15–3.07)
Pooled(M-H)							1.81	(1.25–2.63)

Q = 8.10, p = 0.920, I^2^ = 0.0%.

**Table 2:** Case-control studies of Factor V Leiden Polymorphism and All Preeclampsia.

study	country	ethnicity	cases		controls		OR	95%CI
			GG	GA/AA	GG	GA/AA		
Lindoff(1997)	Sweden	Caucasian	39	11	45	5	2.54	(0.81–7.93)
Mimuro(1998)a	Australia	83% Caucasian	46	4	149	1	12.96	(1.41–118.83)
De Groot(1999)	Netherland	Caucasian	147	16	148	15	1.07	(0.51–2.25)
Mello(1999)	Italy	Caucasian	34	12	77	3	9.06	(2.40–34.18)
O’Shaughnessy(1999)	UK	Caucasian	268	15	94	6	0.88	(0.33–2.33)
Kim(2001)	US	Caucasian	235	15	241	12	1.28	(0.59–2.80)
D’Elia(2002)	Italy	Caucasian	55	3	71	3	1.29	(0.25–6.64)
Morrison(2002)	Scotland	Caucasian	377	17	155	8	0.87	(0.37–2.07)
Prochazka(2003)	Czech	Caucasian	34	4	47	3	1.84	(0.39–8.78)
Fabbro(2003)	Italy	Caucasian	49	3	76	4	1.16	(0.25–5.42)
Prasmusinto(2004)	Germany/Croatia	Caucasian	36	4	71	1	7.79	(0.85–73.20)
Faisel(2004)	Finland	Caucasian	126	7	108	4	1.50	(0.43–5.26)
Driul(2005)	Italy	American Indian	35	4	62	2	3.54	(0.62–20.33)
Davalos(2005)	Mexican	Caucasian	31	2	60	2	1.94	(0.26–14.41)
Mello(2005)	Italy	Caucasian	389	13	389	13	1.00	(0.46–2.18)
Demir(2006)	Turkey	Caucasian	60	10	97	5	3.23	(1.05–9.92)
Yalinkaya(2006)	Turkey	Caucasian	94	6	96	4	1.53	(0.42–5.60)
Hiltunen(2009)	Finland	Caucasian	238	10	663	16	1.74	(0.78–3.89)
Best(2009)	US	American Indian	82	5	159	6	1.62	(0.48–5.60)
Kahn(2009)	Canada	Caucasian	107	6	421	22	1.07	(0.42–2.71)
Seremak-Mrozikiewicz(2010)	Poland	Caucasian	99	10	386	14	2.78	(1.20–6.46)
Dissanayaka(2012)	Sri Lanka	Caucasian	168	7	169	2	3.52	(0.72–17.20)
Malek-Khosravi(2012)	Western Iran	Caucasian	183	15	93	8	0.95	(0.39–2.33)
Pooled(M-H)							1.60	(1.28–2.00)

Q = 26.02, p = 0.251, I^2^ = 15.5%.

a: 83% of the cases were Caucasian and 17% were Asian.

**Table 3:** Case-control studies of Prothrombin 20120 G-A Polymorphism and Severe Preeclampsia.

study	country	ethnicity	cases		controls		OR	95%CI
			GG	GA/AA	GG	GA/AA		
Kupferminc(1999)	Israel	Caucasian	32	2	107	3	2.23	(0.36–13.93)
Kupferminc(2000)	Israel	Caucasian	58	5	122	4	2.63	(0.68–10.16)
Kupferminc(2000)	Israel	Caucasian	50	5	151	5	3.02	(0.84–10.86)
Livingston(2001)a	US	60% African American	110	0	96	1	0.29	(0.01–7.23)
Alfirevic(2001)b	UK	90% Caucasian	61	2	42	2	0.69	(0.09–5.08)
Tempfer(2004)	Austria	Caucasian	23	1	23	1	1.00	(0.06–16.97)
Koleva(2005)	Bulgaria	Caucasian	36	3	99	4	2.06	(0.44–9.67)
Gerhardt(2005)	German	Caucasian	93	4	271	6	1.94	(0.54–7.04)
Mello(2005)	Italy	Caucasian	362	44	398	8	6.05	(2.81–13.02)
Demir(2006)	Turkey	Caucasian	29	5	95	7	2.34	(0.69–7.93)
Seremak-Mrozikiewicz 2010)	Poland	Caucasian	63	5	395	5	6.27	(1.76–22.28)
Malek-Khosravi(2012)	Western Iran	Caucasian	68	2	98	3	0.96	(0.16–5.90)
Pooled(M-H)							3.02	(2.06–4.45)

Q = 11.65, p = 0.39, I^2^ = 5.8%.

a: 60% of the cases were African American, 40% were white Caucasian;

b: 90% of the cases were Caucasian and 10% were non-Caucasian.

**Table 4:** Case-control studies of Factor V Leiden Polymorphism and Severe Preeclampsia.

study	country	ethnicity	cases		controls		OR	95%CI
			GG	GA/AA	GG	GA/AA		
Dizon-Townson(1996)a	US	94% Caucasian	144	14	386	17	2.21	(1.06–4.59)
Kupferminc(1999)	Israel	Caucasian	25	9	103	7	5.30	(1.80–15.60)
van Pampus(1999)	Netherland	Caucasian	267	16	66	1	4.20	(0.55–32.15)
Rigo(2000)	US	Caucasian	98	22	98	3	7.33	(2.13–25.30)
Tempelhoff(2000)	Germany	Caucasian	23	6	58	3	5.04	(1.16–21.88)
Kupferminc(2000)	Israel	Caucasian	48	15	118	8	4.61	(1.83–11.58)
Livingston(2001)b	US	60% AfricanAmerican	105	5	94	3	1.49	(0.35–6.41)
Alfirevic(2001)c	UK	90% Caucasian	62	1	41	3	0.22	(0.02–2.19)
Kim(2001)	US	Caucasian	158	11	241	12	1.40	(0.60–3.25)
Currie(2002)	Australia	Caucasian	42	4	40	6	0.64	(0.17–2.42)
Tempfer(2004)	Austria	Caucasian	21	3	22	2	1.57	(0.24–10.36)
Koleva(2005)	Bulgaria	Caucasian	29	10	97	6	5.58	(1.87–16.64)
Gerhardt(2005)	German	Caucasian	90	7	255	22	0.90	(0.37–2.18)
Mello(2005)	Italy	Caucasian	338	68	391	15	5.24	(2.94–9.34)
Demir(2006)	Turkey	Caucasian	28	6	97	5	4.16	(1.18–14.64)
Seremak-Mrozikiewicz(2010)	Poland	Caucasian	62	6	386	14	2.67	(0.99–7.20)
Malek-Khosravi(2012)	Western Iran	Caucasian	66	4	93	8	0.70	(0.20–2.44)
Pooled(D-L)							2.45	(1.63–3.69)

Q = 36.45, p = 0.003, I^2^ = 56.1%.

a: 94% of the cases were white Caucasian, 3% Hispanic, 2% Asian, 0.8% African and 0.4% Native American.

b: 60% of the cases were African American, 40% were white Caucasian;

c: 90% of the cases were Caucasian and 10% were non-Caucasian.

**Figure 2:**
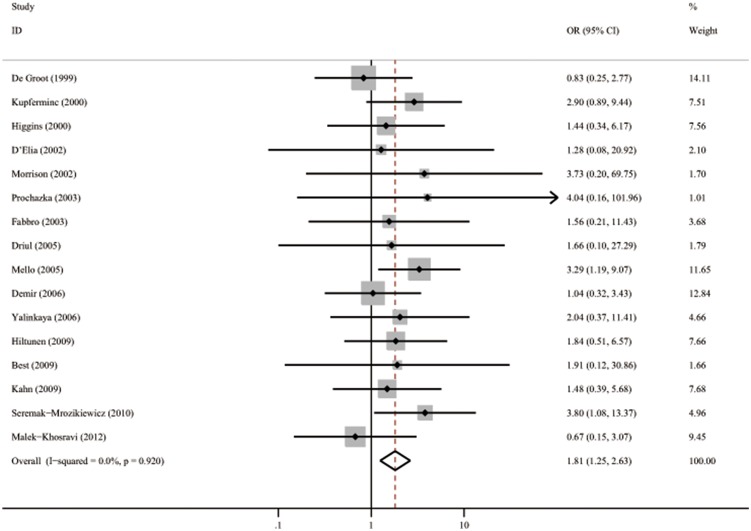
Forest plot of OR with 95%CI of all preeclampsia associated with the prothrombin G20210A polymorphism by fixed model. Black square means value of OR, and the size of the square means inversely proportional to its variance. Horizontal line means 95% confience interval (CI) of OR. Black diamond means pooled results.

**Figure 3:**
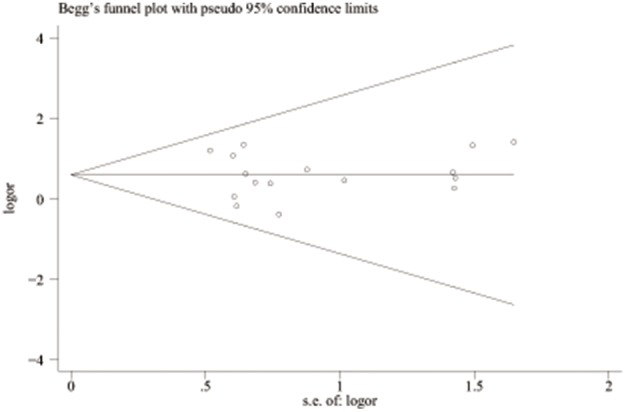
Begg’s funnel plot of publication bias test of all preeclampsia associated with the prothrombin G20210A polymorphism. LogOR means nature logarithm of OR (odds ratio). Horizontal line means the summary estimate, while the sloping lines mean the expected 95% confidence interval.

Table 1 lists the 16 studies (2296 cases and 3262 controls) that evaluated the association between PT 20210 polymorphism and all preeclampsia [9], [10], [18]–[21], [26], [27], [33], [35], [42]–[47]. The pooled OR was 1.81 (95%CI 1.25–2.63) with a *Q* statistic for heterogeneity of 8.10 (*p* = 0.920). No heterogeneity was found (*I* ^2^ = 0.0%), and the fixed model was used. These results are shown graphically in Figure 2. Figure 3 is the funnel plot showing the distribution of the individual measures of these studies. No publication bias was suggested by the funnel plot.

**Figure 4:**
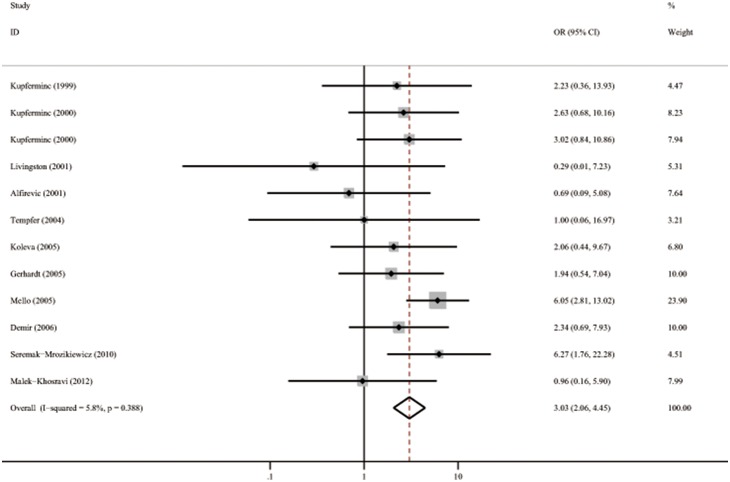
Forest plots of OR with 95%CI of severe preeclampsia associated with the prothrombin G20210A polymorphism in fixed model. Black square means value of OR, and the size of the square means inversely proportional to its variance. Horizontal line means 95% confience interval (CI) of OR. Black diamond means pooled results.

**Figure 5:**
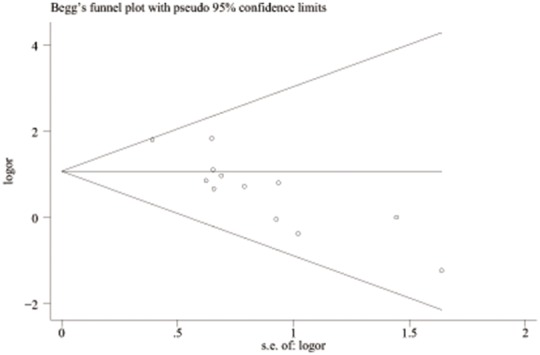
Begg’s funnel plot of publication bias test of severe preeclampsia associated with the prothrombin G20210A polymorphism. LogOR means nature logarithm of OR (odds ratio). Horizontal line means the summary estimate, while the sloping lines mean the expected 95% confidence interval.

The effect of the PT 20210 polymorphism and severe preeclampsia is shown in Table 3. There are 12 studies involving a total of 1063 cases and 1946 controls[9], [10], [16]–[18], [22], [25], [33], [35], [38], [48], [49]. Only two studies [9], [10] reported a significant association between this polymorphism and severe preeclampsia. The pooled OR was 3.02 (95%CI 2.06–4.45) with *p* heterogeneity of 0.390. The results are shown in Figure 4. The funnel plot suggests a potential publication bias (Figure 5). Influence analyses, in which pooled OR are calculated omitting one study at a time, demonstrated that the results were influenced by Mello’s study [10] to some extent. The pooled OR decreased significantly from 3.02 (95%CI 2.06–4.45) to 2.08 (95%CI 1.31–3.32) after the omission of this study.

**Figure 6:**
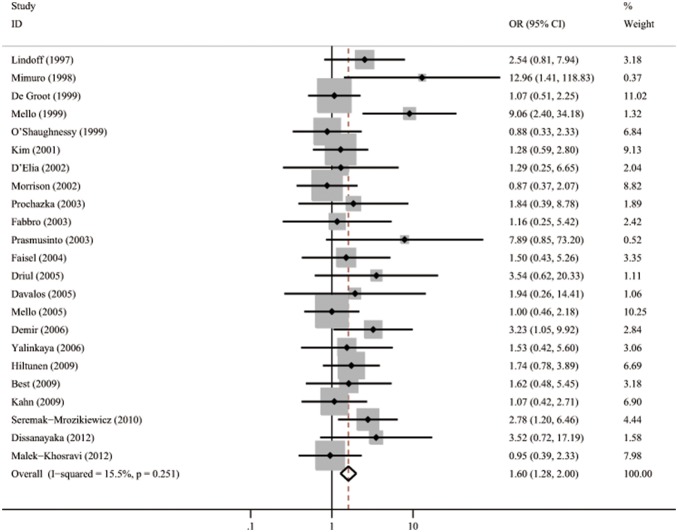
Forest plots of OR with 95%CI of all preeclampsia associated with FVL in fixed model. Black square means value of OR, and the size of the square means inversely proportional to its variance. Horizontal line means 95% confience interval (CI) of OR. Black diamond means pooled result.

**Figure 7:**
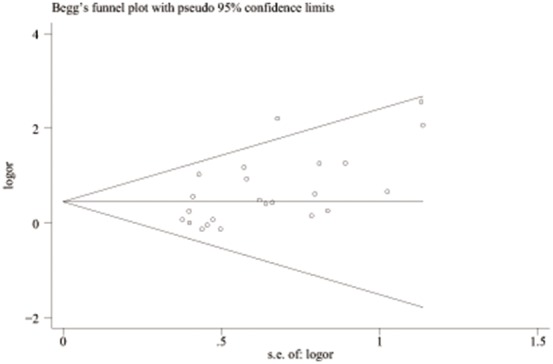
Begg’s funnel plot of publication bias test of all preeclampsia associated with FVL. LogOR means nature logarithm of OR (odds ratio). Horizontal line means the summary estimate, while the sloping lines mean the expected 95% confidence interval.

Table 2 summarizes the analysis of 23 studies involving 3131 patients and 4036 controls that assessed the association between FVL and all preeclampsia [7]–[10], [19]–[21], [23], [26], [27], [29], [30], [32], [33], [35], [41], [42], [44]–[47], [50], [51]. Meta-analysis revealed a significant association between FVL and preeclampsia (pooled OR = 1.60, 95% CI 1.28–2.00, *p* = 0.251, *I* ^2^ = 15.5%) when a fixed model was applied (Figure 6). The funnel plot shows obvious evidence of publication bias in the lack of small negative studies (Figure 7).

**Figure 8:**
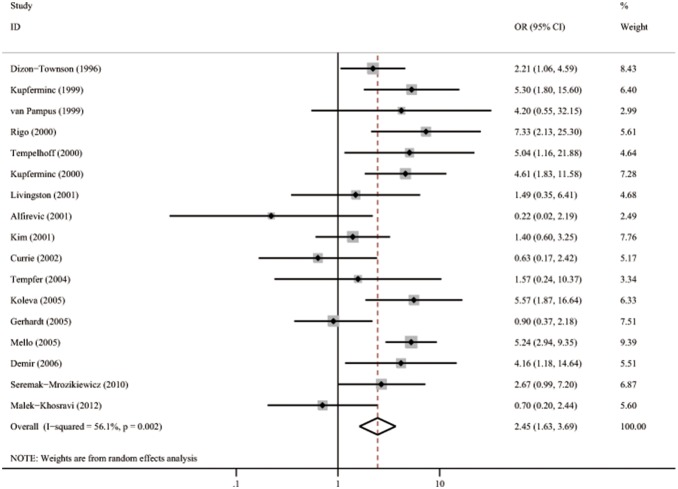
Forest plots of OR with 95%CI of severe preeclampsia associated with FVL in random model. Black square means value of OR, and the size of the square means inversely proportional to its variance. Horizontal line means 95% confience interval (CI) of OR. Black diamond means pooled results.

**Figure 9:**
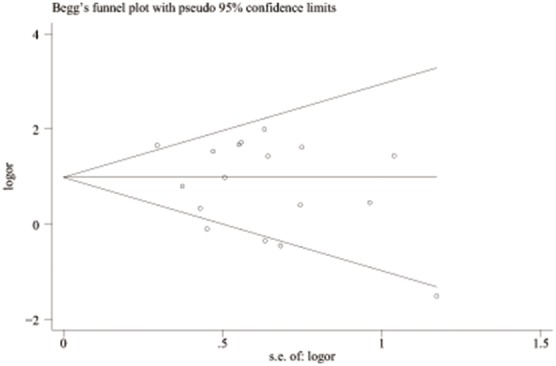
Begg’s funnel plot of publication bias test of severe preeclampsia associated with FVL. LogOR means nature logarithm of OR (odds ratio). Horizontal line means the summary estimate, while the sloping lines mean the expected 95% confidence interval.

Seventeen studies with 1814 patients and 2721 controls evaluated the association between FVL and severe preeclampsia, as shown in Table 4 [9], [10], [15]–[17], [22], [25], [32], [33], [35], [37]–[40], [48], [49], [52]. Obvious evidence of heterogeneity (*Q* = 36.45, *p* = 0.003, *I* ^2^ = 56.1%) was found between studies. Thus, a random model was used for which the pooled OR was 2.45 (95% CI 1.63–3.69). The results are shown graphically in Figure 8. Figure 9 is the funnel-plot analysis, which seems to be asymmetric. However, Egger’s test suggests no obvious evidence of publication bias (Egger’s test p = 0.246).

## Discussion

Preeclampsia is multifactorial gestational vascular complication that greatly threatens the life of pregnant women and their fetuses. The etiology of preeclampsia remains complex. Early studies reported that thrombophilia genes were associated with a hypercoagulable state [5], [6], which may partly explain the development of preeclampsia. In recent decades, great attention has been paid to the role that thrombophilia genes may play in the development of preeclampsia. The aim of this meta-analysis was to evaluate the association between FVL or prothrombin G20210A polymorphism and preeclampsia.

In our meta-analysis, a 2-fold increased risk of preeclampsia and an approximately 3-fold increase for severe preeclampsia are observed when the prothrombin 20210A allele exists. After the exclusion of one study [10] the pooled OR for severe preeclampsia decreased markedly, although it was still statistically significant. The patients in this study seemed to have more severe disease with acute complications in comparison with other studies. After omission, the pooled OR decreased to 2.08, close to the pooled OR for all preeclampsia. This finding demonstrates that the roles played by prothrombin G20210A SNP in the increased risk of preeclampsia and severe preeclampsia may have no difference. Subgroup analyses of ethnicity were not conducted because of the lack of studies involving patients with genetic backgrounds other than Caucasian, which limited broadening the interpretation of our results to the general population.

These results are inconsistent with those of Lin and August [12], which revealed no significant associations. In Lin’s meta-analysis, a relatively small sample size (916 cases for all preeclampsia and 325 cases for severe preeclampsia) was collected in comparison with ours. It is suggested that the lack of statistical significance in the former meta-analysis might be due to the smaller number of studies. Therefore, our meta-analysis with a larger sample size (2296 cases for all preeclampsia and 1063 cases for severe preeclampsia) should give a more precise estimate under greater statistical power.

Our meta-analysis also indicates that FVL is associated with an increased risk of preeclampsia. The combined OR for FVL associated with all preeclampsia was 1.60 (95%CI 1.28–2.00), and the *Q*-statistic test suggested a mild heterogeneity between studies. The funnel plot was asymmetric, and the Egger test suggested obvious evidence of small-study bias. These results are similar to those of former meta-analyses [12], [53], and help to confirm that FVL may increase the risk of preeclampsia.

When the studies assessing the relationship between FVL and severe preeclampsia were involved in meta-analysis, there was a 2.45-fold increase in risk. High heterogeneity was found among all studies (*I* ^2^ = 56.1%, *p* = 0.003). This unexplained heterogeneity may be due to the wide variation of the prevalence of the FVL in the control populations. In most studies, the prevalence of FVL in the control group ranged from 2% to 5%, including all of the larger studies in this meta-analysis, All of them reported ORs much greater than 1. On the contrary, seven studies [16], [17], [22], [25], [35], [37], [48] had a higher prevalence of FVL in control groups (6.3%–13%), four of which reported ORs less than 1. After excluding the studies with a high FVL prevalence in control groups, the heterogeneity clearly decreased (*I* ^2^ = 25.1%, *p* = 0.212 versus *I* ^2^ = 56.1%, *p* = 0.003). The funnel plot showed no significant evidence of publication bias.

There are some limitations to our meta-analysis. First, the recruitment of patients and controls varied in different studies. In some studies the cases were matched with controls in several factors that may confound, e.g., age, weeks of gestation at delivery, systolic and diastolic blood pressure at <20 weeks’ gestation, and protein C deficiency, whereas in others they were not. These confounding factors may have added some bias to our analysis, although because the definition of preeclampsia and severe preeclampsia varied little in these studies, the selection bias would not be large. Second, because the majority of patients and controls come from the Caucasian population, our conclusion may not be applied to other populations and the general population. Although one study [28] has assessed this association in an Asian population, no conclusion was drawn due to the absence of FVL polymorphism in both case and control groups. It seems that this polymorphism is rarely detected in Asian population. Third, a moderate to high heterogeneity existed between the studies evaluating the association between FVL and severe preeclampsia, for which ethnic background explained little. Even though the heterogeneity decreased sharply after excluding studies with high FVL prevalence in control groups, it had nothing to do with the interpretation of results. More details are needed to analyze the source of heterogeneity. Last, there was some evidence of publication bias (i.e., lack of small negative studies) in our analysis. A larger sample size is essential for thorough meta-analysis, and more studies with small numbers of cases and controls recruited under the same inclusion criteria can help to decrease the publication bias.

In conclusion, these two thrombophilia gene polymorphisms, FVL and prothrombin G20210A, are associated with an increased risk of preeclampsia and severe preeclampsia, respectively. A large number of cases and controls are required to make a more precise risk estimate and minimize the bias in meta-analysis.

## Supporting Information

Checklist S1 **PRISMA Checklist.**
(DOC)
